# Using an Alternative Method to Estimate Overcount for Census 2021.

**DOI:** 10.23889/ijpds.v7i3.1903

**Published:** 2022-08-25

**Authors:** Zoe White, Johanna Hall, Kristina Xhaferaj

**Affiliations:** 1 Office for National Statistics

## Objectives

The project aimed to test an alternative method to estimate the number of duplicate responses in the 2021 England and Wales Census. The method utilises information from all census records instead of relying on samples. It requires less clerical review than the original inverse sampling method used for overcount estimation.

## Approach

We used the Splink implementation of Fellegi-Sunter to match the 2021 Census to itself. The resulting linked dataset was filtered to retain only the top scoring record pair for each unique census record, giving a final dataset of around 71.5 million record pairs. These pairs were divided by score into 13 homogeneous buckets. Random samples of 1000 pairs per bucket were clerically reviewed to determine whether each pair was a true duplicate or not. The clerical results were used to assign an estimated probability of being a duplicate to each bucket and hence to every census record within the bucket.

## Results

A dashboard was created which contained percentages of duplicates by region and ‘overcount group’ for the original and alternative methods. This enabled us to view the data side by side and create visualisations to aid analysis. The alternative method had higher average duplicate percentages in overcount groups for communal establishments and was also higher for 9 out of 10 regions in the armed forces overcount group. We found that both the original and alternative methods of overcount estimation followed the same pattern in terms of minimum and maximum duplicate percentages except for those in communal establishments where minimum and maximums were both higher than the original method. Additionally, the minimum was marginally higher for the student and armed forces overcount groups for the alternative method.

## Conclusion

On initial comparison, estimated rates of duplication resulting from the different methods are comparable. We plan to conduct further analysis on the similarities and differences of the two methods, and research whether the new method could be applied to estimate the duplication rate in other large datasets including administrative data.

